# Evaluation of User Interface and Workflow Design of a Bedside Nursing Clinical Decision Support System

**DOI:** 10.2196/ijmr.2402

**Published:** 2013-01-31

**Authors:** Michael Juntao Yuan, George Mike Finley, Ju Long, Christy Mills, Ron Kim Johnson

**Affiliations:** ^1^Ringful HealthAustin, TXUnited States; ^2^CHRISTUS St Michael's Health SystemTexarkana, TXUnited States; ^3^Texas State University - San MarcosDepartment of Computer Information SystemsSan Marcos, TXUnited States

**Keywords:** clinical decision support systems, user-computer interface, software design, human computer interaction, usability testing, heuristic evaluations, software performance, patient-centered care

## Abstract

**Background:**

Clinical decision support systems (CDSS) are important tools to improve health care outcomes and reduce preventable medical adverse events. However, the effectiveness and success of CDSS depend on their implementation context and usability in complex health care settings. As a result, usability design and validation, especially in real world clinical settings, are crucial aspects of successful CDSS implementations.

**Objective:**

Our objective was to develop a novel CDSS to help frontline nurses better manage critical symptom changes in hospitalized patients, hence reducing preventable failure to rescue cases. A robust user interface and implementation strategy that fit into existing workflows was key for the success of the CDSS.

**Methods:**

Guided by a formal usability evaluation framework, UFuRT (user, function, representation, and task analysis), we developed a high-level specification of the product that captures key usability requirements and is flexible to implement. We interviewed users of the proposed CDSS to identify requirements, listed functions, and operations the system must perform. We then designed visual and workflow representations of the product to perform the operations. The user interface and workflow design were evaluated via heuristic and end user performance evaluation. The heuristic evaluation was done after the first prototype, and its results were incorporated into the product before the end user evaluation was conducted. First, we recruited 4 evaluators with strong domain expertise to study the initial prototype. Heuristic violations were coded and rated for severity. Second, after development of the system, we assembled a panel of nurses, consisting of 3 licensed vocational nurses and 7 registered nurses, to evaluate the user interface and workflow via simulated use cases. We recorded whether each session was successfully completed and its completion time. Each nurse was asked to use the National Aeronautics and Space Administration (NASA) Task Load Index to self-evaluate the amount of cognitive and physical burden associated with using the device.

**Results:**

A total of 83 heuristic violations were identified in the studies. The distribution of the heuristic violations and their average severity are reported. The nurse evaluators successfully completed all 30 sessions of the performance evaluations. All nurses were able to use the device after a single training session. On average, the nurses took 111 seconds (SD 30 seconds) to complete the simulated task. The NASA Task Load Index results indicated that the work overhead on the nurses was low. In fact, most of the burden measures were consistent with zero. The only potentially significant burden was temporal demand, which was consistent with the primary use case of the tool.

**Conclusions:**

The evaluation has shown that our design was functional and met the requirements demanded by the nurses’ tight schedules and heavy workloads. The user interface embedded in the tool provided compelling utility to the nurse with minimal distraction.

## Introduction

### Usability Issues in Clinical Decision Support Systems

Clinical decision support systems (CDSS) are important tools to improve health care outcomes and reduce preventable medical adverse events [1,2]. In the US, CDSS is one of the key requirements for the government mandated meaningful use of electronic medical record (EMR) adoption [3]. It was suggested that smart, portable, point-of-care, and interoperable technology solutions could help reduce inefficiencies and improve patient safety and outcomes for nurses [4].

However, the effectiveness and success of CDSS depend on their implementation context and usability in complex health care settings (eg, [5]). Studies have shown that different CDSS implementations often yield very different clinical outcomes (eg, [6,7]). A study found that a home grown CDSS designed specifically for a hospital out-performed 31 other similar CDSS deployments included in the study [8]. A multi-site study indicated that nurses routinely over-ride CDSS recommendations that do not fit their local practice, leading to a potential increase of errors [9].

In particular, CDSS implementations often suffer from poor usability, which directly impacts their adoption and effectiveness. For instance, user interface (UI) workarounds have been shown to greatly diminish the effectiveness of widely used CDSSs [10,11]. While many CDSSs rely on alert/reminder-based user interactions to prompt the clinician correct potential guideline violations, alert fatigue was a common issue for those systems (eg, [12]). A study showed that physicians who receive CDSS alerts were only slightly more likely to take appropriate actions than those who do not [13]. In the area of diagnostic decision support, it has been demonstrated that the accuracy of diagnostic aid tools depends on their UI. Tools that require simple copying and pasting from free text medical records yield more accurate results than tools that require the physician to extract and categorize information from the medical records [14,15]. As a result, usability design and validation, especially in real world clinical settings, are crucial aspects of successful CDSS implementation.

In this study, we developed a novel CDSS for the CHRISTUS St. Michael health system (a 350 bed acute care hospital) to help frontline nurses better manage critical symptom changes in hospitalized patients. The CDSS is currently undergoing clinical pilots inside the hospital. The goal of the CDSS was to reduce preventable failure to rescue (FTR) cases in the hospital. Since the nursing work environment is subject to constant interruptions and is error prone [16], a robust UI and implementation strategy that fit into the existing workflow was crucial to the success of the system.

In this paper, we will discuss the design, evaluation, implementation, and validation of the CDSS UI. We will present several innovations in nursing CDSS UI design, especially on large touch screen devices. The internal algorithmic design and the validation of decision rules, however, are beyond the scope of this paper. In the next section, we will start with a brief clinical background of the nursing CDSS tool.

### Nursing Decision Support for Early Detection of Critical Changes

#### Early Symptom Recognition and Response

The FTR is a leading patient safety indicator with the highest incident rates among all indicators according to a recent large-scale study [17]. In 2010, FTR measure was included as one of the Inpatient Prospective Payment System measures by the Center for Medicare and Medicaid Services, which directly affects hospitals’ reimbursements [18].

FTRs are often considered preventable because the symptoms of a deteriorating patient could present hours before the rescue starts. Examples of such critical symptom change include patient complaint of a new pain, mental status change, and difficulty breathing etc. Studies have indicated that many FTRs could have been averted if the critical symptoms in patients were captured, evaluated, and communicated early.

It was suggested that the nurses’ early recognition, evaluation, and decision making of symptom signs could play an important role in FTR [19,20]. A study conducted in a surgical oncology population indicated that many complications are detectable by nurses and can be managed with timely intervention [21]. It was suggested that 23,000 in-hospital cardiac arrests in the UK could be prevented every year if early signs of symptoms were detected and acted upon [22]. A 2009 study demonstrated that an early symptom recognition and response system could help improve outcome of sepsis and septic shock, which have hard-to-detect symptoms [23].

Simply detecting and evaluating the critical symptom changes is not enough. The potential complication must be communicated to the rest of the clinical team, and be escalated to the right team members in order to organize effective interventions. It was argued that FTRs are often caused by the failure to communicate [24]. Interventions such as the rapid response team (RRT) have demonstrated effectiveness in reducing FTRs when the issues are escalated on time [25,26]. In fact, the national deployment of RRT has the explicit purpose of supporting nurses in managing critical changes before coding arrest [27]. It was also suggested that escalating to surgical residents could improve rescue success rates [28], indicating that the optimal path of escalation needs to be selected by the nurses as part of the decision-making process.

#### Role of Frontline Nurses in Symptom Evaluations and Rapid Response Interventions

Frontline nurses are often the first to notice critical symptom changes. Their decisions at the point-of-care are crucial factors determining whether FTR events can be reduced. However, at the same time, nurses are ill equipped to manage critical symptom changes in hospitals.

The frontline nursing staff in most hospitals have very high workloads, need to manage extensive multitasking, and are fatigued [16,29]. The fatigue has been demonstrated to negatively impact nurses’ cognitive performance [30], including symptom evaluations. In fact, studies have shown a strong anti-correlation between nursing staffing levels and medical error rates [31].

The average skill and training levels of nurses do not adequately prepare them to evaluate potentially complex symptom changes. A study found that a 10% increase in the proportion of nurses holding a bachelor’s degree was associated with a 5% decrease in the odds of FTR [32]. Furthermore, most diagnostic aid CDSSs, such as differential diagnostic tools and diagnostic reminder tools, were designed for physicians to use in office settings, as opposed to nurses at the bedside.

While the RRT is a proven effective intervention for FTR, RRT resources can be under-utilized [33] because the nurses do not feel comfortable activating the RRT. Better communication has been shown to improve RRT utilization [34]. It has been suggested that mandatory RRT activation helps reduce cardiorespiratory arrests outside of critical care areas in a hospital [35].

The hieratical structure in hospitals is known to impede nurse decision-making process [36]. Nurses are often discouraged from communicating and escalating problems. While hospitals across the nation have implemented teamwork frameworks, such as the TeamSTEPPS [37], the emergency communication between nurses and physicians is still often error prone and require standardization [38].

#### Design of CDSS

A specially designed CDSS could potentially help the nurse address the above issues related to critical symptom changes and FTRs. Such CDSS requires special design considerations for two reasons.

First, the system must be tailored to the nurses’ training and cognitive levels, and generate action items that are appropriate for the nurse. Most floor nurses have gone through less than 4 years of medical training after high school, and they do not have independent authority to treat the patient without the physician’s prescription.

Second, the system must be adapted to the fast paced workflow during a rescue operation. The tool must be ubiquitous, instant on, and provides useful feedback in merely minutes. The application should enhance real-time communication across team members, as opposed to bringing in another computer that impedes face-to-face communication.

Both challenges highlight the need for a novel design, and formal evaluation of the system UI and workflow.

### Cognitive Design of UI

Human-computer interaction and workflow designs are crucial for the success of clinical informatics projects. A large body of research has been devoted to study methods and techniques to evaluate usability of systems.

Early efforts focused on creating human models and breaking down tasks into small pieces that could be directly measured and optimized for user performance. For instance, the goals, operators, methods, and selection rules family of frameworks [39-41] are widely used to model human users as information processors. They break down user actions (eg, every key stroke), and measure time consumed in each step to evaluate the overall effectiveness of the UI. However, such frameworks do not take into account the intrinsic difficulty of the task and the functionality of the UI. They are very good at evaluating systems that predominantly require movement operations, but are less effective in evaluating systems with heavy cognitive tasks.

For cognitive systems, analysis of the UI itself is a key aspect of usability design, because UI design often has a deterministic effect on user performance. Research in cognitive theory has indicated that different visual representation of the same underlying work problem could produce dramatically different user performance in terms of ability to complete tasks correctly and productivity [42,43]. A well-known example is that Arabic numerals are much easier to add and multiply than their equivalent Roman numerals.

Furthermore, complex work often requires collaboration of multiple users. It was demonstrated that cognition can be distributed across multiple users working on the same system [44-46]. Hence, another important aspect of usability design is to evaluate each user’s goals and functions, and then translate them into a cohesive UI.

A popular design approach that works with the above cognitive design principles is the work-centered design (WCD) [47,48]. WCD treats the UI as an aid for the user to achieve a specific work task. It conceptualizes steps for knowledge capture, requirement analysis, aiding design, and evaluation, which is a process followed closely in modern software development.

A particularly interesting application of distributed cognition and WCD in the medical informatics field is the UFuRT (user, function, representation, and task analysis) [49-51] framework. For this project, we decided to use the UFuRT framework as a guide for usability design. The primary reason for us to choose UFuRT is its successful track record in design and evaluation of medical information technology (IT) products [52-54]. Its usability evaluation process consists of 4 major steps:

1. User analysis is used to identify users and stakeholders of the work product, and document their needs and objectives. The user requirements are translated into system design requirements in this process.
2. Function analysis aims to generate an essential description of the work. The UFuRT process calls for a 4-step analysis to detail the dimensions, constraints, relations, and finally operations.
3. Representational analysis is the design process to identify and determine the implementation representations of relations among the dimensions identified in the functional analysis. The representation includes UIs and workflows for different types of users of the system. Representational analysis is a crucial step of the design process since it has been convincingly demonstrated that different representations of the same task can have very different impacts on the user’s efficiency and productivity [55]. The ease-of-use of the UI is also one of the major factors driving adoption of any technology product [56].
4. Task analysis is to identify steps by a specific user on a specific representation in order to carry out an operation.

In the context of our project, we used UFuRT framework to analyze software requirements and inform the specification. Hence, we focused on user analysis and UI design aspects of representation analysis. We performed a high-level functional analysis and did not perform task analysis in the design stage. The reason was that complete functional and task analysis require full knowledge of every detail of the product, which would not provide enough flexibility for our iterative software development process.

## Methods

### Design Goals and System Requirements

The overall objective of the system was to help prevent patient safety events during critical changes. Through interviews with hospital-based clinicians, we have specifically identified symptom evaluation and escalation as the 2 main functional goals of the CDSS.

#### Improve Symptom Recognition and Evaluation

##### Existing Procedures

While nurses do not make diagnoses, they are the first to recognize and evaluate the patient symptom changes. Based on their evaluation, they would decide how to (or whether to) coordinate further care, and their evaluation results are often accepted by the team as the basis of a formal diagnosis.

Existing diagnostic CDSS tools provide a proven framework to help reduce errors in diagnostic evaluation, and improve documentation of the clinical findings that lead to diagnoses. Specially, the CDSS needs to provide 2 core functionalities.

##### Provide Just-in-Time Medical Content to the Nurse

For many critical symptom changes, there are multiple possible diagnoses. An example is that a hospitalized patient suddenly feels chest pain. The chest pain could be an indicator of heart attack, which needs to be attended to by a cardiologist or surgery team immediately; or the chest pain could indicate reflux or indigestion, which is a rather common condition that is simple to treat.

The frontline nurses typically do not have enough medical training and experience to thoroughly evaluate those potential diagnostic outcomes. The CDSS should provide specific instructions for the nurse to follow, and then make recommendations on what to do next. For instance, it should provide specific instructions on whom to call and what to say during the call for each potential diagnosis. The system does not replace human decision-making or training, but it provides support to help nurses deal with complicated emergent situations to the best of their capabilities.

##### Reduce Common Cognitive Errors

Common cognitive errors that lead to diagnostic errors include premature closure, anchoring, confirmatory bias, and framing [57]. Those errors happen because the clinicians ignore certain findings or give certain other findings too much weight. Studies have indicated that cognitive errors such as premature closure are the most common cause of diagnostic errors made by clinicians [58]. A key design goal of the CDSS was to help reduce those common cognitive errors.

To reduce framing and premature closure, the CDSS should encourage and prompt the clinicians to check all possible diagnostic outcomes, especially severe outcomes that lead to FTRs. The CDSS should also prompt the clinicians to verify all important symptoms and findings related to major diagnostic outcomes to minimize missed diagnoses.

To reduce anchoring or confirmatory bias, the CDSS should present an objective estimate of likely diagnoses and suggested clinical actions based on the current findings. The objective probability estimate could reduce the user’s reliance on reconceived decision biases.

#### Facilitate Team Communication

##### Teamwork

Teamwork is one of the few proven approaches to improve patient safety and care quality in hospitals [37,59]. Particularly, our system should be designed to increase the utilization of the RRT, and improve communication between nurses and physicians.

##### RRT Utilization

As we discussed in the clinical background, RRT is an effective approach to help reduce FTR when it is deployed correctly. Our CDSS aimed to improve the effectiveness of the RRT by activating RRT early and making RRT mandatory when the nurse detects certain warning signs.

The CDSS needs to provide an easy and non-intrusive way to automatically alert the RRT at appropriate times. The RRT consists of more experienced clinicians, and they can decide whether or when to respond to those alerts. At the same time, it is important for the CDSS to clearly notify the nurse when it sends alerts to the RRT and the status of the alerts. The user must feel that he/she is in full control in order to effectively utilize the system.

##### Nurse-Physician Communication

If the floor nurse determines that the patient needs assistance from a physician, he/she would call the physician and explain the situation. The conversation could be a frustrating experience for both the nurse and the physician due to different expectations. That could result in the physician losing confidence in the nursing staff, and nurses delaying calls to physicians. The system should provide tools to help nurses communicate better with physicians in emergency situations.

### Development of the Software Specification

#### Design

We used the UFuRT framework as a conceptual guide to develop the software specification for the CDSS tool. Specifically, we identified users of the system, and documented use case stories for each user (ie, user analysis). We identified high-level functions the system must perform to meet the user requirement (ie, functional analysis). And finally, we created visual representations of the UI that can best accomplish those functions (ie, representational analysis). The UFuRT task analysis was not conducted at the design stage. Instead, the tasks were evaluated as part of the user evaluation process described later in this article.

#### User Analysis

Users of the proposed CDSS were members of the clinician team responsible for rescuing patients in the hospital. They included floor nurses, RRT nurses, and physicians. The user roles described in this section were based on interviews with hospital clinicians.

The primary users of the CDSS were the floor nurses. The system presented information and actions that were appropriate to the floor nurses. Specifically, the system could not present medical content that required MD-level training to understand, or ask nurses to make diagnostic decisions on their own. The CDSS also could not instruct the nurse to perform clinical actions that he/she was not authorized or qualified to do, such as performing advanced examinations, ordering labs, or writing prescriptions. Furthermore, a key characteristic in the floor nurse’s work environment is that they are very busy and have established workflows. The system added minimal overhead to the existing workflows.

If the floor nurse detected a potential problem, the RRT nurse was the next escalation step. RRT nurses are typically paged by the hospital internal communication system, and hence the CDSS must support paging the RRT. The system should give RRT nurses more options as they have the authority to perform standing orders on patients. Finally, when the RRT nurse arrived at the bedside, in order to minimize errors at the hand-off of care, it was important for the CDSS to have clear documentation on the findings and actions that have been performed by the floor nurse so far.

The physician in charge of the patient should be notified when there is a probable problem with the patient. The system should provide accurate and concise summaries of the patient condition for the nurse to read to the physician when talking on the phone.

#### Functional Analysis

Once the user requirements were determined, we developed a list of high-level functions the system must perform. Please note that we did not create a detailed catalog of functions at this stage of development. Instead, we focused on high-level operations in order to provide implementation flexibility. Key operations of the system include the following:

- Identify the symptom change that triggers the use of the system
- Identify a list of potential diagnoses
- Identify a list of potential clinical findings that will reject or affirm those diagnoses
- Enter clinical findings
- Re-evaluate the probabilities for each diagnosis after each finding
- Repeat for all finds until a diagnosis becomes highly likely
- Identify the action items for this diagnosis
- Identify the escalation path for this diagnosis
- Perform operations required in the action items list

In addition, we have also identified non-essential operations that were related to the specific design of the system. Such operations included user login to the system with badge number, synchronization of the device content with online repositories, user entry of the patients’ room number, and user configuration of the device for display options.

#### UI Design

##### Overview

The UI of the product was designed to address operations listed in the previous section. It aimed to present a familiar and non-intrusive interface to the user at the point-of-care. In this section, we describe key features of the UI.

##### Mobility Through a Consumer Tablet Device

We decided to implement the UI on a touch screen consumer tablet device. The reason behind choosing a tablet device was that it can be accessed anytime, anywhere, and could be carried around by the clinician or be made available at the bedside. The tablet device was connected to the hospital secure WiFi system to access medical records, alert RRT and other teams, and update clinical content as needed.

The choice of a consumer tablet, as opposed to a dedicated medical device, was due to two reasons. First, the consumer device was much cheaper to deploy. A consumer iPad costs less than one third of a special purpose tablet PC on the market. Second, the consumer device featured an UI that the nurses were already familiar with due to his or her use of similar devices at home.

The most widely used and user-friendly consumer tablet device on the market is the Apple iPad, which we chose as the implementation platform for the CDSS device.

##### Dynamic Checklist Design

Most existing diagnostic decision support tools use decision trees [60] or text-based free form search [15] to generate potential diagnoses. We determined that neither approach was suitable for nurses in emergence situations. Decision trees are slow and hard to recover from accidental typos. Text-based data entry is very slow on a mobile device.

Instead, we decided to use another UI metaphor that is commonly used in hospital environments—the medical checklist. The main UI of the system was a dynamic checklist for the nurse to go over and examine clinical findings related to the patient. Checklists have been shown to reduce medical errors [61,62], and could help prevent several categories of cognitive errors (outlined in Section 3.1.2 of [63]). UI is important for checklists. Effective checklists need to be prioritized, short, highly usable, and integrated into the clinician workflow [64].

**Figure 1:**
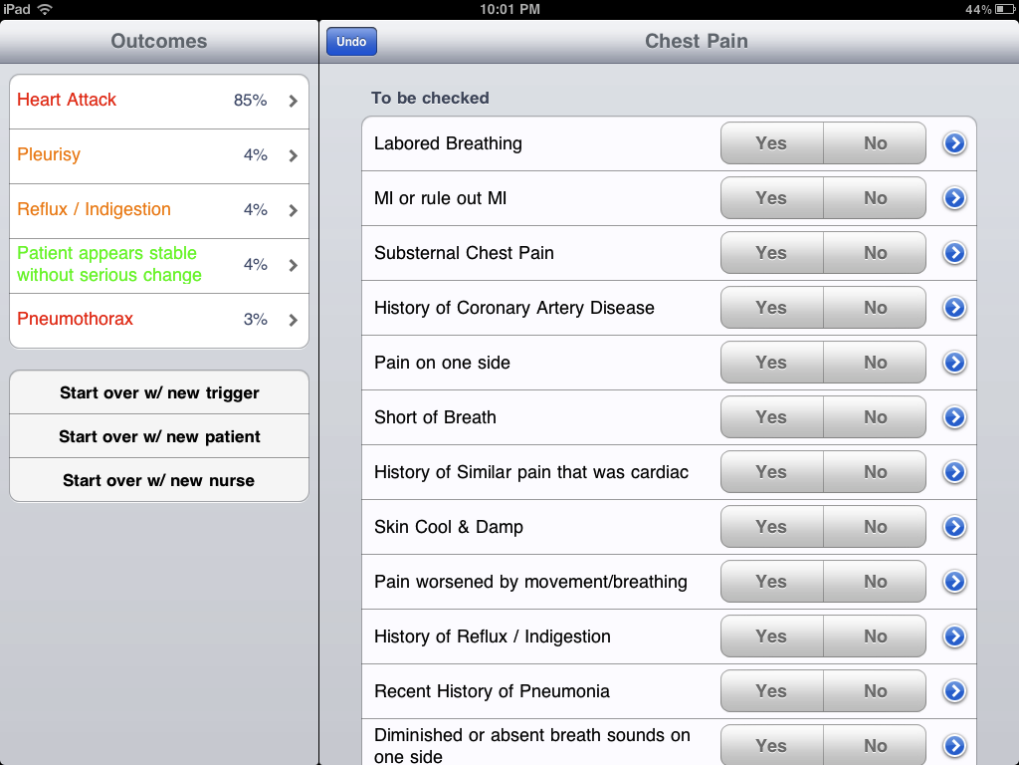
The main split screen user interface of the decision support system.

Figure 1 shows a split panel screen with 2 lists. This is the screen that the nurses see when he/she selects a critical change (eg, "chest pain" or "mental status change"). The checklist to the right is a list of measurements and observations the nurse needs to perform in order to evaluate the patient. The list was ordered based on the priority and potential impact of each finding. The nurses were encouraged to work on the high priority tasks at the top of the list first.

The list on the left shows potential causes for the patient's critical change (ie, the diagnostic outcomes). The causes were listed in order of their probabilities based on the current findings from the checklist items on the right panel.

All the user needed to do was to follow the checklist and enter a simple yes/no answer to the findings. With each yes/no answer, the system automatically recalculated and redisplayed the diagnostic outcome probabilities and the priorities of the remaining checklist items.

The nurses could go through the findings checklist in any order. The nurses could also undo any choices to go back to any previous state. That allowed the nurses to pick and choose tasks that happen to fit the existing workflow at any point of the process. There was no need to interrupt the flow just to provide a finding required by the software.

This is different than the typical decision tree or flow chart decision models, where the workflow is dictated by the software system.

##### RRT Integration

The CDSS was connected to the hospital communication system, and it automatically sent out pages to the RRT as the nurse works on the patient. The RRT members could then decide whether to intervene depending on how severe the patient condition was as reported by the nurse through the device.

If the RRT decided to intervene, they could simply take over the CDSS device, which has documentation of the findings the nurse had already completed.

##### Communication Checklist

**Figure 2:**
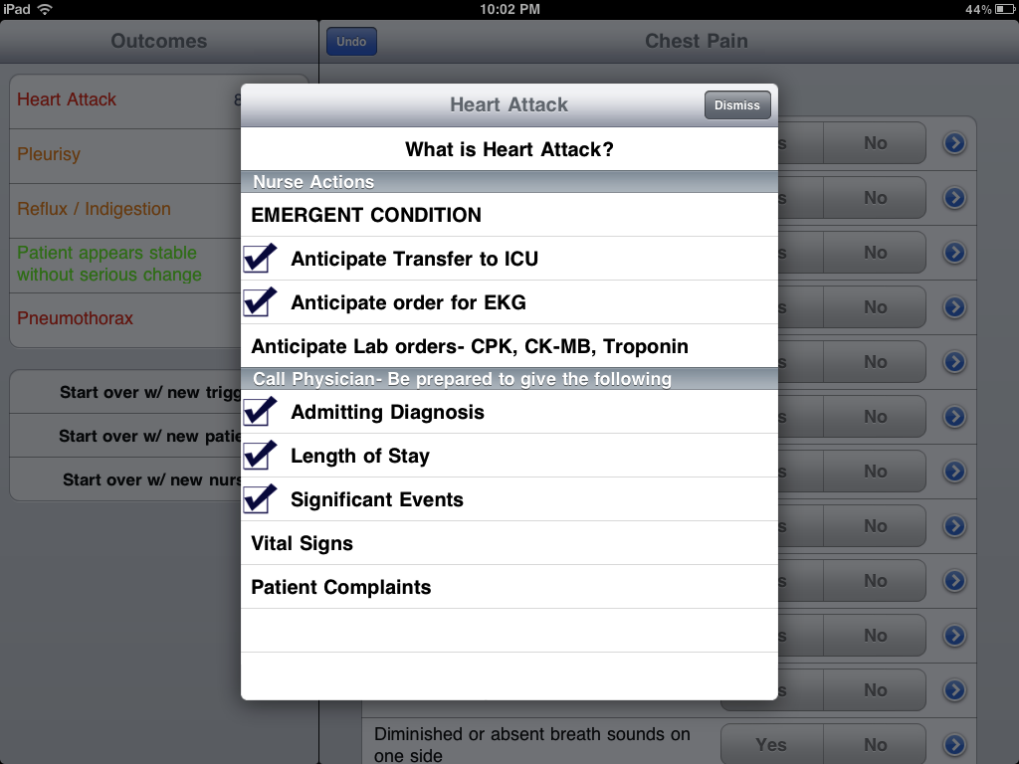
The action items for the nurse after a likely outcome is reached.

The CDSS provided a standard list of items for the nurses to go through with the physicians when a likely diagnosis emerged (Figure 2). The nurse action lists were customized for each diagnostic outcome, and included orders the nurses should anticipate from the physicians. The nurses could get a head start by preparing for those orders while trying to reach the physician, saving time for the patient rescue.

The action items were reviewed and approved by the physicians in the hospital, and they were designed to enable physicians to make quick decisions over the phone.

### Implementation of the CDSS

The CDSS system was implemented as a client-server computer application. The main component of the system was an iPad application developed in Objective C using the Apple iOS software development kit. The iPad application provided all the UI elements described in the design, and it was the only UI device the nurses needed to interact with during the patient evaluation process. The iPad application contained a SQLite-based relational database to store decision rules, medical content, user credentials, and usage logs. The application required access to the hospital’s secure WiFi network in order to send paging messages to the RRT members. Except for the RRT page, the iPad device could function entirely without network connectivity, and only needed to occasionally synchronize with the backend database for content updates.

**Figure 3:**
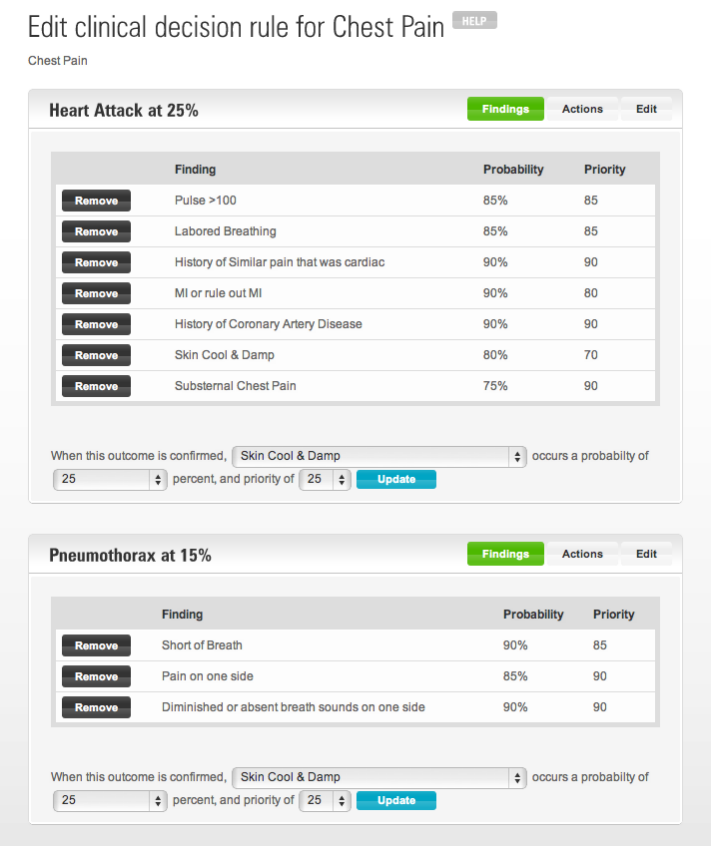
The clinical rule editor in the Web-based CMS.

The second component of the system was an online content management system (CMS) to manage the decision rules, medical contents, and authorized users and devices. The system was designed as a Web application built on Java Enterprise Edition running on Tomcat and MySQL database servers. The interface with the iPad device was programmed as RESTful XML Web services. The CMS had a human UI that visualized the content and allowed CRUD (create, retrieval, update, and delete) operations of the content items from any Web browser. Proper user authorization was enforced in the CMS so that only users with certain roles (eg, physicians and managers) could update the content. Figure 3 shows a screenshot of the CMS Web page that allowed reviewers to associate findings and actions with diagnoses into clinical rules.

The CMS also provided an interface for the physician reviewers to review cases based on the usage log of the iPad device. That supplemented the brief information recorded in formal medical records and provided insights into how to improve the system in the future.

In the next two sections, we will discuss evaluations and validations we performed on the CDSS, especially the iPad UI.

### Evaluation Methods

#### Evaluation Process

The UI and workflow design of the product was evaluated using heuristic evaluation and performance-based end user evaluation. The heuristic evaluation was done after the first prototype, and its results were incorporated into the product before the performance-based evaluation was conducted.

#### Heuristic Evaluation

Heuristic evaluation is a formal UI evaluation method designed to uncover potential problems in a product [65-68]. It is particularly well suited for prototype and early stage products as a discounted alternative to full usability testing [68]. A heuristic study is typically conducted by 3-5 independent expert evaluators who are trained on UIs. Studies have suggested that 3 expert evaluators can uncover 80-90% of usability problems that would have been uncovered by a full usability study from end users [69]. In health care IT, heuristic evaluation has been successfully used to evaluate UIs for products ranging from EMRs [70] to medical devices [68,71].

In this project, we incorporated heuristic evaluation into the iterative product design and development process. Based on the functional requirements outlined earlier in this paper, we built a first prototype, conducted heuristic evaluation, and then improved the prototype by addressing the heuristic violations identified by the evaluators.

It was demonstrated that the evaluators who are experts in both UI design and the specific application domain tend to be most effective in identifying heuristic violations [69]. Since a key requirement in our product was to cause minimal disruption to the clinical workflow, we believed that evaluators with strong domain expertise are crucial. We recruited 4 evaluators to study the initial prototype. JL is an information scientist trained in usability evaluation and technology adoption. She is an associate professor at the Texas State University. CM is a registered nurse and hospital quality management specialist. She has over 5 years of experience with RRTs in hospitals. She received training by JL to conduct heuristic evaluation. RM is a registered nurse of 20 years of experience with 5 years in the RRT. She received training from JL to conduct heuristic evaluation. CE is a registered nurse of 15 years of experience with 5 years in the RRT. He received training from JL to conduct heuristic evaluation.

The evaluators went through all UI elements in the application, and used the 10 heuristics in the computer software for evaluation [65]. The heuristic violations were coded and documented. They were then rated for severity by all evaluators in the team. The severity was rated on the scale of 0 to 4, where a score of 0 meant that it is not a usability problem at all, 1 was a cosmetic problem only that did not need to be fixed unless extra time was available, 2 was a minor usability problem and fixing this was given low priority, 3 was a major usability problem that was important to fix and was given high priority, and 4 was related to release block issues and was imperative to fix before the product could be released.

The heuristic violations were entered into an issue tracking system for the engineering team. The product reached its first release after all heuristic violations rated 3 and above were fixed.

#### Performance-Based Evaluation

##### Overview

Once the first release of system was developed, we assembled a panel of nurses to evaluate the UI and workflow via simulated use cases. The panel consisted of 10 nurses from our target user group in the hospital. The panelists had varied education background and experience levels. There were 3 licensed vocational nurses and 7 registered nurses on the panel. All of them were non-rapid response nurses working full time on the floor. Their work experience ranged from 1 to 39 years, with a median of 23 years. The simulation study was conducted as follows.

1. The nurse enters a patient room to meet the study monitor. The monitor gives a trigger symptom verbally to the nurse.
2. The nurse goes back to the station and fetches the tablet device. On the way, he/she will enter badge number, room number, and select the trigger symptom from a list.
3. When the nurse enters the room again, he/she can go through the checklist in any order. The nurse will verbally ask the monitor questions on the checklist, and the monitor will provide a yes/no answer.
4. When the nurse has received enough information, he/she decides on a likely diagnostic outcome for the patient.
5. The nurse will read out aloud each of the action item associated with the diagnostic outcome.

The process was repeated 3 times for each nurse. The tablet device automatically logged usage during the sessions.

##### Task Completion

We recorded whether each nurse successfully completed each session. The first session for each nurse was considered a training session to get the nurse familiar with the device, and was not included in the evaluation results. The success criterion was to have the nurse walkthrough the entire process and reach the action items without external help.

##### Completion Time Evaluation

For each session, we recorded the entire duration from the time the nurse walked into the room to the point where the nurse finished reading the action items. The completion time was an estimate of how much overhead time the use of the device added to the whole workflow. Since the product was designed to help nurses make quick decisions in urgent situations, it was crucial that the tool does not introduce too much overhead on its own. The evaluation criterion for the tool was that it should add less than 5 minutes of overhead to the existing clinical workflows.

##### NASA Task Load Index

After each session, the nurse was asked to use the National Aeronautics and Space Administration (NASA) Task Load index [72] to self evaluate the amount of cognitive and physical burden associated with using the device. The NASA task load index is a validated instrument for evaluating the burden of multiple tasks a user has to perform in parallel. It is well suited for the use scenario of this application where the user is required to multitask. The NASA Task Load Index has been successfully applied in evaluating health care IT products in the past [73]. The evaluation criterion for the released product was that the task load introduced by the tool should be minimal.

## Results

### Key Issues Identified in Heuristic Evaluation

In Table 1, we list a few examples of the heuristic violations identified by the evaluators. Each issue was categorized into one of the 10 common software application heuristics [65], identified by the place in the software product where it occurs, and assigned a severity based on the consensus rating by the evaluators.

A total of 83 heuristic violations were identified in the studies. Tables 2 to 4 list the distribution of the heuristic violations and their average severity.

The released version of the product had all heuristic violations rated 3 and above fixed. In this study, heuristic evaluation conducted by experts improved the usability of the product.

### Performance-Based Evaluation Results

The 10 nurses on the panel successfully completed all 30 sessions of the performance evaluations. All nurses were able to use the device after a single training session with the instructor.

For each nurse, we took the median completion time from the 3 sessions, and then calculated the mean and standard deviation across the 10 nurses. On average, the nurses took 111 seconds (SD 30 seconds) to complete the simulated task. That is well within the 5 minutes overhead goal that we had set.

The NASA Task Load Index results indicated that the work overhead on the nurses was low. In fact, most of the burden measures were consistent with zero, as seen in Table 5. The only potentially significant burden was temporal demand, which is consistent with the primary use case of the tool. The tool was designed for the nurses to go over the symptom and vital signs checklists quickly, hence it exerts natural temporal pressure to its users.

**Table 1:** Example heuristic violations.

Heuristics violated	Place of occurrence	Severity	Usability problem description
Visibility of system status	Start	3.8	When syncing the application, there was no way to know if it will take 15 seconds or 10 minutes. It would be nice to know that it will take approximately 1 minute or show a percent completion.
Match between system and the real world	Outcome	3.4	List the outcomes as percentages instead of just a number without percentages.
User control and freedom	Checklist	4	The user should have the ability to change an answer once it has gone down to the list of answered questions. I can see frustration with the process if you have to completely start over to change an answer.
Consistency and standards	Outcome	1	Color code should be far apart along the visible spectrum so that the outcome can be clearly distinguished.
Error prevention	Checklist	4	Have the user confirmation when backing out of a screen that would cause the user to have to reenter all data.
Recognition rather than recall	Checklist	2	Abbreviations are used in the checklist. It should follow a simple primary rule.
Flexibility and efficiency of use	Checklist	3	If we add future triggers, there needs to be a way to ensure that when the keyboard displays that it does not cover the last triggers. Currently it is not a problem but should build this into system now.
Aesthetic and minimalist design	Outcome	3	There were too many "start over" displays currently. It would be simpler to have 1 button with a drop down screen listing the options: trigger, patient, or user. The questions also need to be reviewed by Dr. Finley and the RRT as currently there are a few questions that ask the same thing, but are just worded differently, and duplicating the questions is unnecessary.
Help user Recognize, diagnose, and recover from errors	Start	4	When a user accidentally hit the home button on iPad, the system will close without any warning and all data will be lost. Restarting within 1 minute allows you to get back to where you were. Otherwise the program will close.
Documentation and help	Outcome	3	The outcomes are in different colors. I am not sure that the staff will know what the color-coding means. Define the color scheme.

**Table 2:** Number of the heuristic violations across the heuristics.

Heuristics violated	Count of usability problem description
Aesthetic and minimalist design	4
Consistency and standards	10
Documentation and Help	13
Error prevention	6
Flexibility and efficiency of use	4
Help user recognize, diagnose, and recover from errors	12
Match between system and the real world	10
Recognition rather than recall	4
User control and freedom	8
Visibility of system status	12
Grand total	83

**Table 3:** Severity of the heuristic violations.

Heuristics violated	Average of severity
Aesthetic and minimalist design	2.25
Consistency and standards	1.49
Documentation and Help	3.01
Error prevention	3.88
Flexibility and efficiency of use	2.88
Help user recognize, diagnose, and recover from errors	2.48
Match between system and the real world	2.50
Recognition rather than recall	2.20
User control and freedom	3.13
Visibility of system status	2.93

**Table 4:** Places of the heuristic violations occurrence.

Places of occurrence	Count of heuristics violated
Action	13
Checklist	33
Outcome	13
Start	24
Grand total	83

**Table 5:** The task burdens measured by the NASA Task Load Index.

Task burden	Average out of 100 (SD)
Mental demand	10.0 (7.4)
Physical demand	1.8 (2.1)
Temporal demand	20.4 (24.8)
Performance	10.7 (11.3)
Effort	4.5 (4.9)
Frustration	1.6 (2.5)

## Discussion

We have demonstrated that the usability of the CDSS is suitable for nurses in hospital environments. However, the ultimate success of the CDSS tool depends on many factors beyond usability, such as training and culture. In the next phase of the project, we have received generous funding from the Center for Medicare and Medicaid Innovations and CHRISTUS Health System to deploy the CDSS in 17 acute and long care facilities in a 3-year clinical deployment. The direct measurement of FTR cases and preventable complications at the deployment sites will provide the ultimate validation of the efficacy of the tool in improving patient safety and hospital care.

In this paper, we discussed the UI design and evaluation of a new decision support tool for nurses. The system was designed to help nurses recognize and escalate early warning signs of patient deterioration in acute care settings. The system will be used by floor nurses to evaluate patients on a daily basis. It will automatically alert the RRT when probable diagnoses are reached.

Using established cognitive design framework UFuRT as a guide, we were able to identify key requirements for the product, create a high-level functional specification, and then translate those functions into UI designs. During the implementation of the product, we performed heuristic evaluation to iteratively identify 83 usability issues, and fixed all issues rated as severe. These design and implementation approaches can be widely used in many different types of software development projects.

After the product was developed, we validated the design by performing end user usability tests, including performance tests and NASA Task Load Index evaluation. The evaluation has shown that our design was functional and met the requirements demanded by the nurses’ tight schedules and heavy workloads.

UI design and implementation were critical factors contributing to successful deployment of the CDSS tools, but they were not the only factors. In follow-up research, we will deploy the solution in a working hospital environment, and evaluate the clinical outcome measures to determine the barriers and efficacy of the overall solution.

## Abbreviations

CDSS: clinical decision support systems
CMS: content management system
EMR: electronic medical record
FTR: failure to rescue
IT: information technology
NASA: National Aeronautics and Space Administration
RRT: rapid response team
UFuRT: user, function, representation, and task analysis
UI: user interface
WCD: work-centered design
